# Lights from the Dark: Neural Responses from a Blind Visual Hemifield

**DOI:** 10.3389/fnins.2017.00290

**Published:** 2017-05-23

**Authors:** Alice Bollini, Javier Sanchez-Lopez, Silvia Savazzi, Carlo A. Marzi

**Affiliations:** ^1^Department of Neuroscience, Biomedicine and Movement, University of VeronaVerona, Italy; ^2^National Institute of NeuroscienceVerona, Italy

**Keywords:** blindsight, perceptual awareness, event related potential, hemianopia

## Abstract

Here we present evidence that a hemianopic patient with a lesion of the left primary visual cortex (V1) showed an unconscious above-chance orientation discrimination with moving rather than static visual gratings presented to the blind hemifield. The patient did not report any perceptual experience of the stimulus features except for a feeling that something appeared in the blind hemifield. Interestingly, in the lesioned left hemisphere, following stimulus presentation to the blind hemifield, we found an event-related potential (ERP) N1 component at a post-stimulus onset latency of 180–260 ms and a source generator in the left BA 19. In contrast, we did not find evidence of the early visual components C1 and P1 and of the later component P300. A positive component (P2a) was recorded between 250 and 320 ms after stimulus onset frontally in both hemispheres. Finally, in the time range 320–440 ms there was a negative peak in right posterior electrodes that was present only for the moving condition. In sum, there were two noteworthy results: Behaviorally, we found evidence of above chance unconscious (blindsight) orientation discrimination with moving but not static stimuli. Physiologically, in contrast to previous studies, we found reliable ERP components elicited by stimuli presented to the blind hemifield at various electrode locations and latencies that are likely to index either the perceptual report of the patient (N1 and P2a) or, the above-chance unconscious performance with moving stimuli as is the case of the posterior ERP negative component. This late component can be considered as the neural correlate of a kind of blindsight enabling feature discrimination only when stimuli are moving and that is subserved by the intact right hemisphere through interhemispheric transfer.

## Introduction

The search for the neural correlates of visual consciousness is undoubtedly one of the most exciting and challenging enterprises of cognitive neuroscience (see Panagiotaropoulos et al., 2014). Currently, there are two basic approaches to tackle this challenge: One is to study healthy participants with visual stimuli rendered invisible by means of various psychological or psychophysical procedures, such for example visual masking or subliminal stimulation. The crucial strategy here is to compare the neural response to the same stimuli when yielding conscious vs. unconscious performance, see Schmid and Maier (2015), for a recent review. The other approach is to find out what are the cognitive and neural mechanisms that enable some patients with cortical blindness to perform above chance in various visual tasks despite lack of perceptual awareness. This approach was pioneered by Poeppel et al. (1973) and Weiskrantz et al. (1974) who demonstrated that stimuli presented to the blind hemifield of hemianopic patients could be reliably spatially located either with saccadic or manual pointing movements despite lack of perceptual awareness. Following these initial findings, a vast series of studies has provided precious information on the functions that can be carried out without perceptual awareness, a phenomenon termed “blindsight” by Weiskrantz et al. (1974). Even though some findings have been questioned (see Cowey, 2010), the bulk of the results provides robust evidence, see reviews by Weiskrantz (2004, 2009), Danckert and Rossetti (2005) and Tamietto and Morrone (2016), of the existence of this phenomenon and of its relevance for trying to select out mechanisms related to the shift from unconscious to conscious perception.

Both the above approaches have yielded key information for understanding the limits and the capacities of unconscious vision, that is, to what extent cognitive functions depend on perceptual awareness, see recent evidence in healthy participants by Koivisto and Rientamo (2016). However, it is the latter (neural) approach that is obviously better suited to enable a search of the neural structures involved in the shift from unconscious to conscious vision. In particular, important evidence has been gathered by means of various brain imaging techniques (see Urbanski et al., 2014 for a general review) such as functional magnetic resonance imaging (fMRI) (Martin et al., 2012; Barleben et al., 2015; Ajina et al., 2015a,b,c) or event related potentials (ERPs), see Railo et al. (2011) for a review. Moreover, further important information has been provided by behavioral studies in blindsight patients with either selective cortical lesions (see for a recent review Chokron et al., 2016) or hemispherectomy (Tomaiuolo et al., 1997; Ptito and Leh, 2007; Leh et al., 2010; Georgy et al., 2016). Finally, interesting evidence has been provided by studies of blindsight in non-human primates (Stoerig and Cowey, 1997; Leopold, 2012; Schmid and Maier, 2015) recently including marmosets (see review by Hagan et al., 2016).

All that said, one of the main unanswered questions concerns the temporal aspects of the processing of unconscious with respect to conscious visual information. That is, at what processing stage and at what corresponding neural level does perceptual awareness emerge? Clearly, fMRI is not ideally suited for answering this question given its relatively low temporal resolution. In contrast, non-invasive electrophysiological techniques such as electroencephalography (EEG), and in particular ERP, with its optimal temporal resolution constitute an invaluable tool that we have used in the present study.

From a theoretical point of view there are two main positions on the time of emergence of perceptual awareness: On one side, there are theories positing an early activation of the visual cortex as a crucial site, such as, for example, the Recurrent Processing (RP) theory of Lamme (2010). On the other side, there are theories positing a later activation in fronto-parietal areas, such as for example the global workspace theory (GWT) proposed by Dehaene and Naccache (2001). Both theories are somewhat controversial: For example, it has been shown that some patients with V1 lesion could still report some form of awareness especially with fast-motion stimuli (Barbur et al., 1993; Ffytche et al., 1996; Milner, 1998; Ffytche and Zeki, 2011) or with TMS stimulation of the intraparietal sulcus (IPS) (Mazzi et al., 2014; Bagattini et al., 2015) and this is not in keeping with the RP theory. However, it should be noticed that whether this form of awareness is visual or not is still debated and difficult to demonstrate, see for example Macpherson (2015). By the same token, also the GWT has received some criticism, for example as a result of the findings of a negative ERP component recorded around 200 ms post stimulus onset, i.e., in N1 domain, over posterior cortical areas that correlates with different degrees of visual awareness (Koivisto and Grassini, 2016; Tagliabue et al., 2016; for review see Koch et al., 2016).

To try and further explore the problems raised by the above controversial picture, in the present study we focused on assessing whether and at what latency stimuli presented to the blind hemifield of hemianopic patients can elicit visually evoked responses that might correlate with the presence of blindsight or residual conscious vision. ERP studies of blindsight are rather scanty: There have been some attempts, with contrasting results, to find reliable ERP responses following blind hemifield stimulation, see Kavcic et al. (2015) for a review. In a pioneering paper, Shefrin et al. (1988), found in one hemianopic patient with blindsight a P300 component when a target word was presented to the blind field. However, interestingly, no P100 was found in this as well as in the hemianopics without blindsight tested. In Kavcic et al. (2015) study there was no evidence of reliable behavioral response to moving dots presented to the blind hemifield and no evidence of ERP response in the damaged hemisphere. However, they found that the damaged hemisphere could be activated via interhemispheric transfer from the intact hemisphere. Importantly, this was the case only in left brain-damaged patients suggesting that the right hemisphere has a special ability to transfer visual motion information to the other hemisphere, see behavioral evidence for this possibility in Marzi et al. (1991). At any rate, apart from possible transfer asymmetries, Kavcic et al.'s results show that the presence of viable callosal or extracallosal connections between intact and damaged hemisphere is of key importance for understanding the mechanisms of plastic reorganization possibly leading to partial or total restoration of vision (see discussion in Celeghin et al., 2015b).

In the present study, we tested two hemianopic patients with a V1 lesion as well as healthy participants in an orientation discrimination of moving or static visual gratings while recording ERPs. We found an interesting relationship between behavioral performance, subjective report, and electrophysiological responses which provides novel information on timing and site of emergence of a sort of rudimental perceptual awareness.

## Materials and methods

### Participants

#### Healthy participants

Eight healthy participants (3 males, 27 ± 6 years old) were tested as visually intact controls.

All were right-handed with normal or corrected-to-normal vision and with no history of neurological or cognitive disorders.

#### Hemianopic patients

##### Patient LF

**Figure 1:**
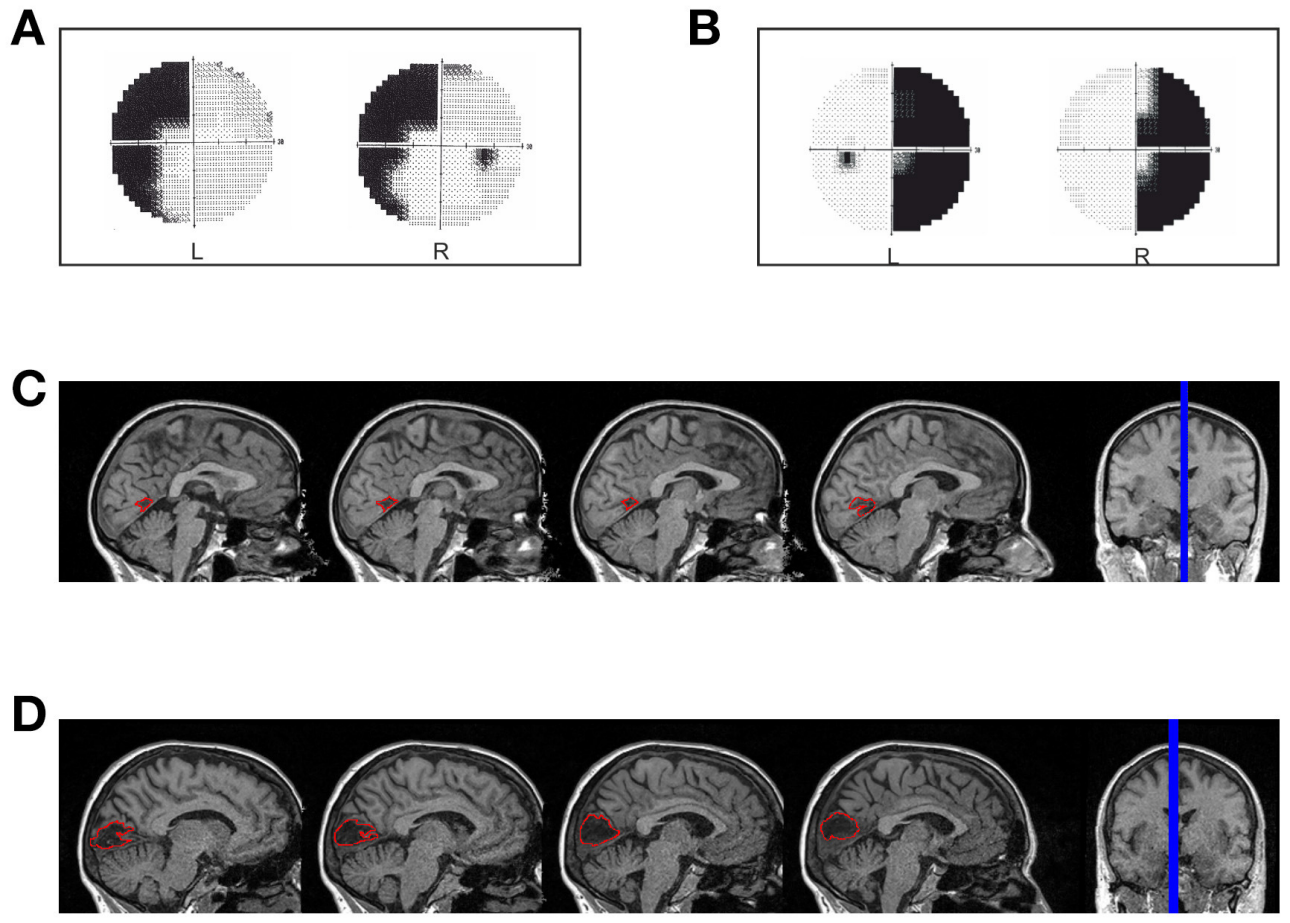
**Hemianopic Patients: (A)** Visual field defect in patient LF; **(B)** Visual field defect in patient SL; **(C)** reconstruction of the lesion in patient LF; **(D)** reconstruction of the lesion in patient SL.

LF (female, 49 years old, right-handed) has a left superior quadrantanopia (Figure 1A) as a consequence of an ischemic stroke. The lesion involves the cortex of the anterior half of the right calcarine fissure up to the origin of the parieto-occipital fissure (Figure 1C). The patient was tested 30 months after the ischemic event.

##### Patient SL

SL (female, 47 years old, right-handed) has a right homonymous hemianopia with partial foveal and upper hemifield sparing (Figure 1B) as a consequence of an ischemic stroke with hemorrhagic evolution. The lesion involves the median para-sagittal portion of the left occipital lobe, with peri-calcarine fissure distribution (Figure 1D). The patient was tested 69 months after the event.

Healthy participants and patients signed an informed consent to participate in the study as well as to their personal information be anonymously published. The study was approved by the Ethics Committee of the Azienda Ospedaliera Universitaria Integrata of Verona and of the ERC and conducted in accordance with the 2012-13 Declaration of Helsinki.

### Behavioral procedure and statistical analysis

Healthy and brain-damaged participants were tested in a light-dimmed room. They were comfortably seated in front of a 24-inch LCD monitor (ASUS VG248) with a refresh rate of 144 Hz driven by a PC used for stimulus presentation. The stimuli were black and white square-wave gratings of 4° of visual angle with a Michelson contrast of 100% against a gray background of 18.33 cd/m^2^ and a spatial frequency of 0.8750 c/°. The gratings' mean luminance was 29.46 cd/m^2.^. They could have either a vertical (0°) or horizontal (90°) orientation and could be static or moving (apparent motion), vertical gratings drifting rightward and horizontal gratings drifting downward. Stimuli were generated using PsychToolBox-3 (Brainard, 1997) running on Matlab^1^. The retinal eccentricity of stimulus presentation used for healthy controls was 9° measured from the inner portion of the display to the central fixation point along the horizontal meridian and 7° along the vertical meridian in the upper visual field, while for hemianopic patients the eccentricity varied according to the field defect (Patient LF: 13° horizontally and 7° vertically; Patient SL: 16° horizontally, 7° vertically).

**Figure 2:**
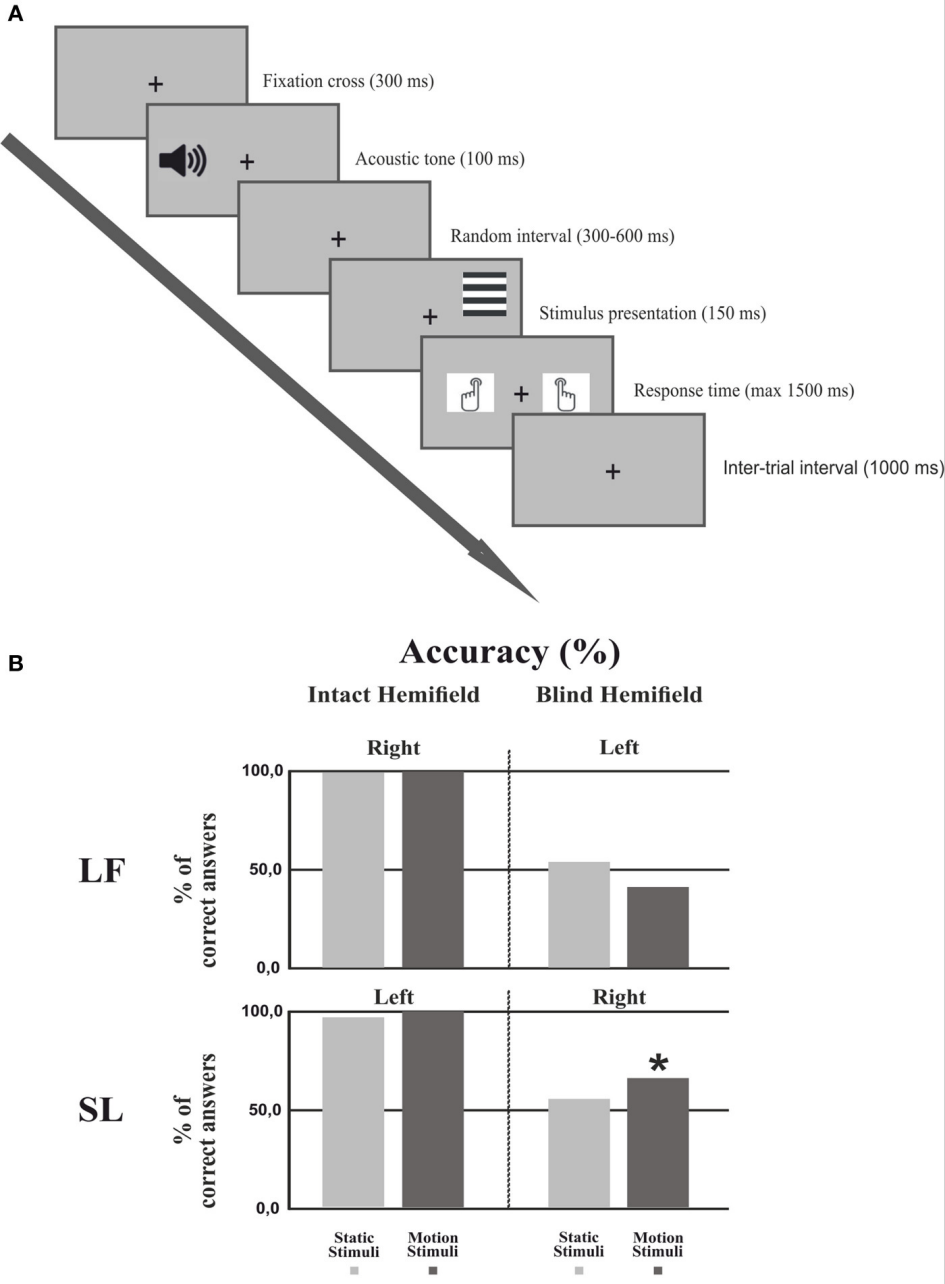
**Experimental procedure and Behavioral results. (A)** Experimental procedure: First, a fixation cross was presented for 300 ms followed by an acoustic tone lasting 100 ms. After a random interval (300–600 ms) the stimulus was presented for 150 ms. The subject had 1500 ms to respond by pressing a keyboard button. The inter-trial interval lasted 1,000 ms. **(B)** Behavioral results: Percentage of correct responses in each hemifield for each condition in both patients. The asterisk indicates that the number of correct responses in the motion condition was significantly different from the chance level of 50%.

The behavioral paradigm (Figure 2A) consisted of four different trial blocks repeated four times (960 trials) and alternating in the following order: Static gratings in the right and then in the left field, moving gratings in the right and then in the left field. In patients the sequence started from the intact field, a block consisted of 60 trials of vertical or horizontal gratings presented in random order; in 30% of the trials no stimuli were presented (catch trials). Participants were asked to perform an orientation discrimination task regardless of whether the stimuli were moving or static. Trials started when a fixation cross of 0.15° appeared in the center of the screen for 300 ms, followed by an acoustic tone (1,000 Hz). After a random interval (300–600 ms) the stimulus was presented to the left or right hemifield for 150 ms and participants had 1,500 ms to press as quickly as possible one of two keyboard keys, using the right or the left index finger to signal a vertical or horizontal grating, respectively (counterbalanced across subjects). The inter-trial interval was 1,000 ms. Importantly, patients were asked to press one of the two keys also when they did not perceive any stimulus in the blind hemifield (including catch trials).

For statistical analysis we used a two-tailed binomial test which in patients allowed to assess if performance in the blind hemifield was significantly higher than chance level (50%). Two binomial tests were performed, one for the motion condition and one for the static condition.

### EEG recording and analysis

EEG activity was continuously recorded from 64 active electrodes (actiCap, Brain Products GmbH, Munich Germany) placed according to the 10-10 International System and was acquired in one experimental session with BrainAmp (Brain Products GmbH, Munich, Germany) and BrainVision software. All scalp electrodes were referenced online to the left mastoid and re-referenced offline to the arithmetically derived average of left and right mastoids. The ground electrode was placed at AFz position. Additionally, horizontal and vertical eye movements were recorded with four electrodes placed at the left and right canthi and above and below the right eye, respectively. Impedance was kept below 5 kΩ for each electrode. The EEG was recorded at 1,000 Hz sampling rate with a time constant of 10 s as low cut-off and a high cut-off of 1,000 Hz with a 50 Hz notch filter. The EEG signal was processed offline using a combination of custom scripts written in Matlab^1^ and EEGLAB toolbox (Delorme and Makeig, 2004). Continuous data were bandpass filtered offline between 1 and 100 Hz. The continuous raw data were visually inspected and large signal jumps such as muscle twitches or electrode cable movements were rejected and bad channels were interpolated. Independent component analysis (ICA) decomposition with logistic infomax algorithm Runica (Bell and Sejnowski, 1995; Makeig et al., 1996) was performed (Lee et al., 1999) to separate brain and non-brain source activities. Stereotyped artifacts like blinks were corrected by identification of the corresponding ICs. After artifact correction and source localization, a mean of 24 ICs (STD = 4.83) remained for each subject. Next, data epochs were extracted (from 200 before to 800 ms after stimulus presentation) and baseline corrected (from 200 ms before to stimulus onset). At this point the data were downsampled to 250 Hz. To assess the ERP responses of patients in the blind field and differences among the experimental conditions, a single-case analysis procedure was adopted. Percentile Bootstrap re-sampling (Efron and Tibshirani, 1993) was drawn on each trial of every condition of a patient. The percentile bootstrap method uses surrogate tests which consist of randomly re-sampling with replacement for 5,000 times the original trials among the conditions to create a data distribution from the shuffled data. Surrogate tests have the advantage to make no assumptions about the data. The bootstrap simulation allowed estimation of the patient sampling distribution adapted to any shape suggested by the data, taking into account variance and skewness of the sample. Next, point-by-point ANOVAs or *T*-tests were performed on all channels with the bootstrap data in order to identify differences between catch trials and the moving and static condition. The false discovery rate (FDR) correction (Benjamini and Yekutieli, 2001) was applied to correct for multiple comparisons. In addition, ERP envelope (i.e., minimum and maximum of all electrodes at every time point) was used to calculate which IC gave the largest source contribution to the EEG signals in term of PVAF (percent of variance accounted):

$$\begin{array}{l} PVAF(IC)=100-[100^{*}mean(var(all\_data-back\_proj))/\\ \;meanvar(all\_data)] \end{array}$$

Where “var” stands for variance; “data” refers to EEG signals, as well as the matrix channels x time-points; finally, “back_proj” refers to the ERP activity of the selected IC back-projected to the scalp ERPs (as a forward projection from cortical source to the scalp channels), thus PVAF indicates the contribution of the IC to the ERP (Lee et al., 2015). With this procedure we selected the ICs that maximally accounted for variance at the electrodes. The same procedure was applied for both patients and controls, with the exception that in the control group we used clusters of ICs, which were identified by means of an automated K-means algorithm procedure on scalp maps, ERPs and dipole localizations. In order to better understand the dynamics that underlie the generation of the ERPs in the blind field we used a source reconstruction based on an empirical Bayesian approach. The estimation of the current sources of the ERP components was carried out by using the Statistical Parametric Mapping software (SPM12 of the Wellcome Trust Centre for Neuroimaging, UK). The patient individual T1-weighted structural MRI image was used. The forward computation to prepare the lead field for the subsequent inversions was performed using the boundary element method (EEG-BEM) that create closed meshes of triangles with a limited number of nodes by approximating the compartments that conform the volume conductor (Fuchs et al., 2001). Successively, the inverse solution was computed on the entire ERP period after stimulus onset (i.e., from 0 to 800 ms) by using coherent smooth prior method (COH) (Friston, 2008) smoothness prior similar to LORETA (Pascual-Marqui et al., 2002). Sources in each time window of interest were visualized in terms of maximal intensity projection (MIP) with the corresponding MNI coordinates. Lastly, to compare the EEG data of patients in the intact field with those of healthy controls single-case analyses were performed. The Revised Standardized Difference Test (RSDT) developed by Crawford and Garthwaite (2005) was performed to compare the differences between the patients' scores in the ipsilesional and contralesional electrodes for stimuli presented to the intact and blind hemifield. The mean amplitude of the ERP components was identified by visual inspection and was compared to that of healthy controls. Pair-wise electrodes from left and right hemisphere were selected (F3-F4, F5-F6, FC3-FC4, FC5-FC6, C3-C4, C5-C6, CP3-CP4, CP5-CP6, P3-P4, and P5-P6).

## Results

### Behavior

#### Healthy controls

The performance of healthy controls was accurate and fast: Right static stimuli = 97.3% correct responses and Reaction Time (RT) = 567 ms; right moving stimuli = 98.6%, RT = 554 ms; left static stimuli = 97.8%, RT = 561 ms; left moving stimuli = 98.7%, RT = 554 ms). These data indicate a low task difficulty. There were no statistically significant differences in accuracy or RT between hemifields and between moving and static stimuli.

#### Patients

Figure 2B shows the discrimination accuracy of the two patients in the two hemifields. In the intact hemifield performance was comparable both in accuracy and RT to that of healthy controls (Patient LF: right static = 99.4%, RT = 608 ms; right moving = 99.5%, RT = 552 ms. Patient SL left static = 96.2%, RT = 531 ms; left moving = 99.4%, RT = 577 ms). In the blind hemifield LF performed at chance level with 53.9% (*p* = 0.426) correct responses for static stimuli and 41.06% correct responses for moving stimuli (*p* = 0.089). She did not report any visual sensation upon stimulus presentation. In contrast, SL performed at chance level with static stimuli (55.03%; *p* = 0.400) but with moving stimuli her performance was significantly above chance with 65.56% correct responses (*p* < 0.005). Interestingly, the patient reported “a feeling of something appearing in the blind field” during stimulus presentation without any idea of gratings' orientation or whether they were static or moving.

### EEG

#### Healthy controls

**Figure 3:**
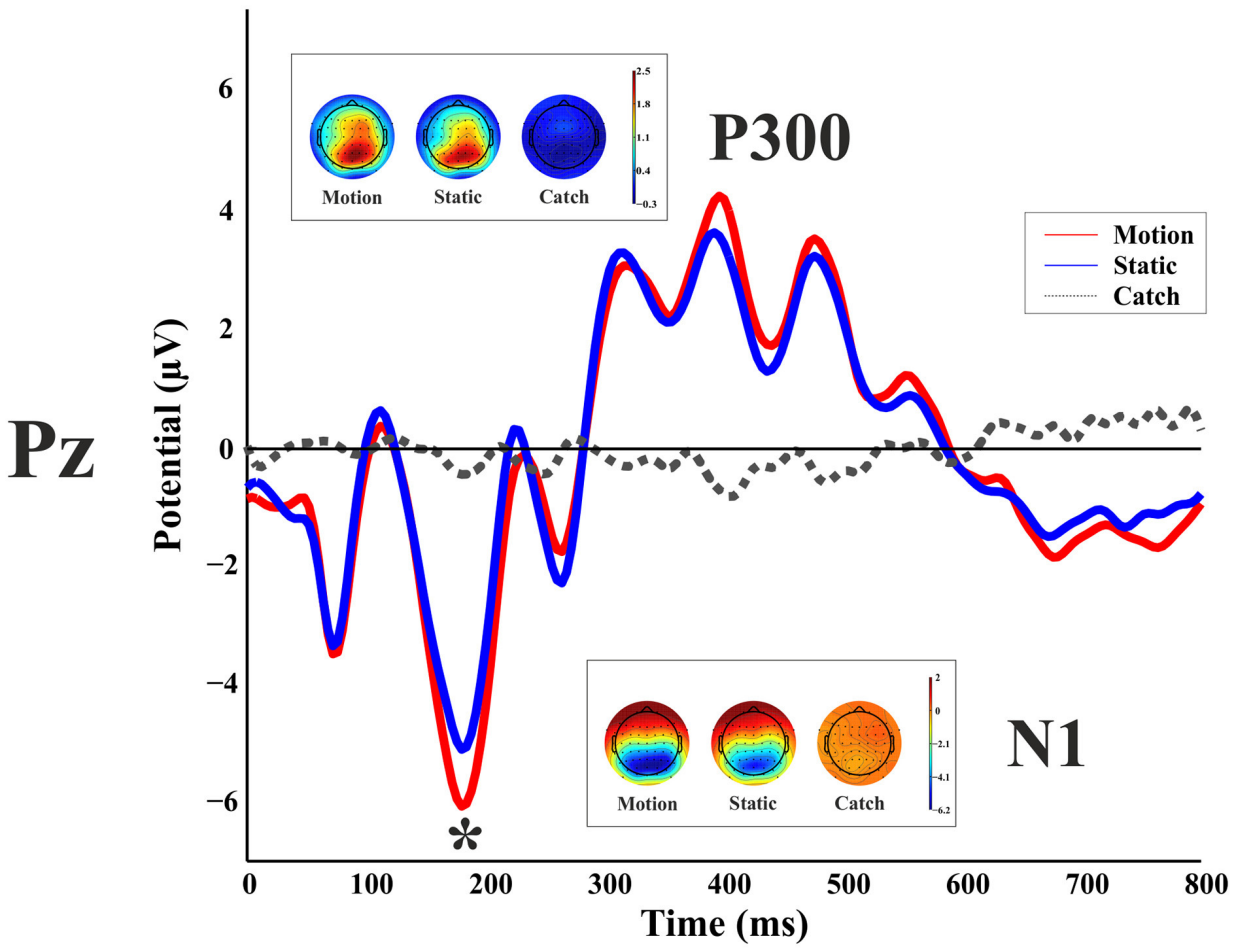
**Healthy participant's grand average ERPs for the motion, static and catch condition as recorded at electrode Pz**. The top and bottom inlets show the topography scalp map for each condition at the time window of N1 **(bottom)** and P300 **(top)**.

Figure 3 shows the ERP responses as recorded at electrode Pz. With a peak detection procedure we found a negative C1 at 75 ms, the sign being in keeping with the site of stimulus presentation in the superior quadrant of the visual field (Jeffreys and Axford, 1972) and therefore with a V1 generator. We found a P1 component at 110 ms; a N1 at 185 ms and a large P3 between 310 and 480 ms. A non-parametric *t*-test on the mean amplitude of the peaks showed a difference between static and moving stimuli only for the N1 component with a larger negative amplitude at the following electrodes: CP6, P1, P2, P4, P6, P7, P8, PO3, PO4, PO8, PO10, O1, Oz, O2 (with a *p*-value < 0.05). These differences were mainly observed in the right hemisphere regardless of the side of visual field of stimulus presentation.

#### Patients

##### LF

**Figure 4:**
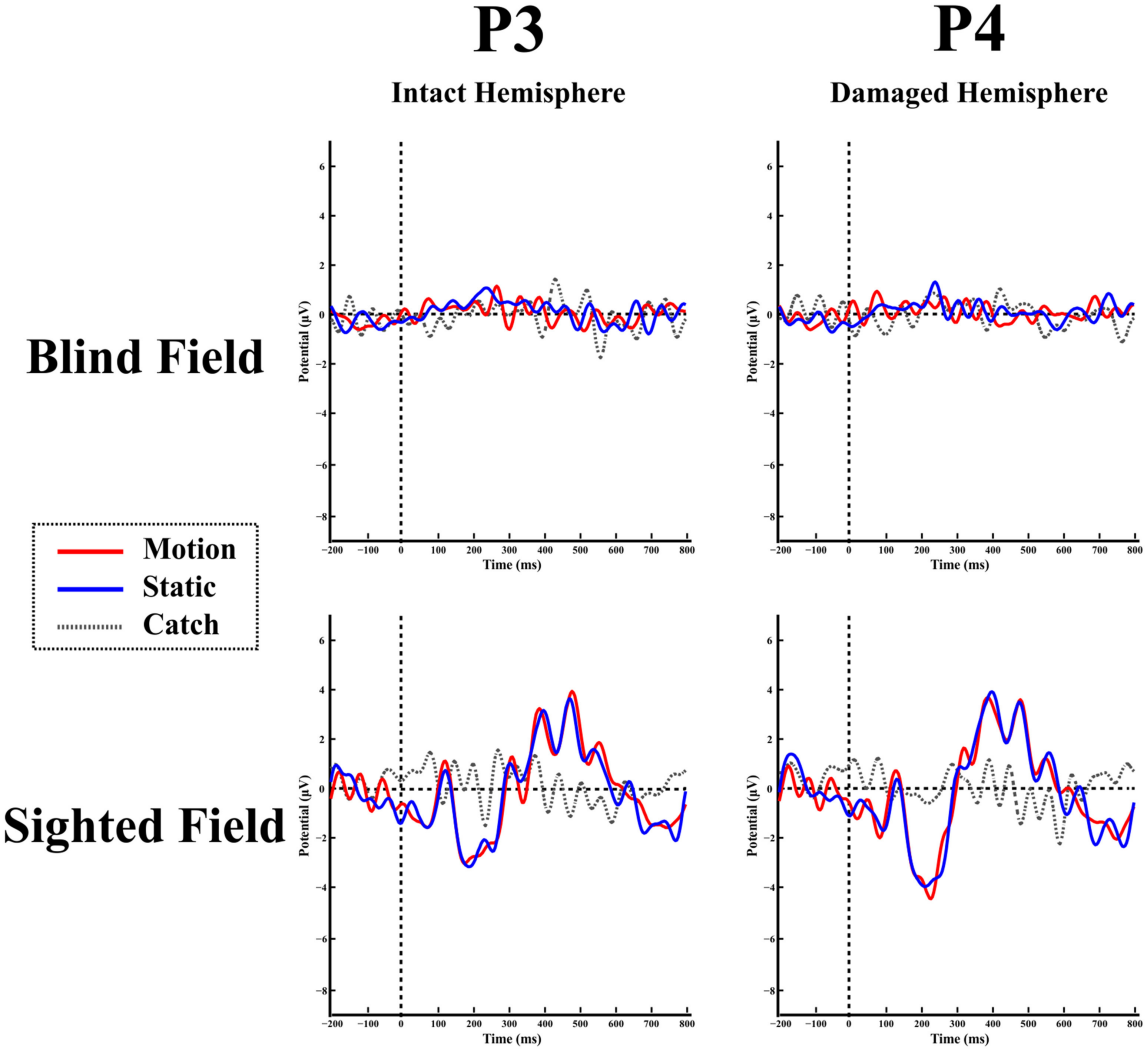
**Patient LF:** ERPs in the blind **(top)** and sighted field **(bottom)** as recorded at P3 (left-intact hemisphere) and P4 (right-damaged hemisphere) for the three stimulation condition.

**Blind hemifield**. Figure 4 shows the ERP responses as recorded at electrode P3 (intact hemisphere) and P4 (damaged hemisphere) for the two hemifields and the three stimulation conditions. As can be seen from Figure 4 there are no reliable ERP responses when the stimulus was presented in the blind field, in keeping with the performance of the patient that was at chance level and without any stimulus-related sensation. A bootstrap ANOVA did not yield any difference between stimulus present and stimulus absent (catch) (all *p*-value were above 0.05 without multiple comparisons correction).

**Intact hemifield**. In contrast, when the stimulus was presented in the intact hemifield ERP responses were similar to those of healthy participants both in latency as well as in amplitude (Figure 4 bottom panel). The differences between responses from left and right hemisphere were similar to those of the control group as demonstrated by the RSDT test where no pair of electrodes yielded reliably different responses with respect to controls (all *p*-value > 0.05).

##### SL

**Figure 5:**
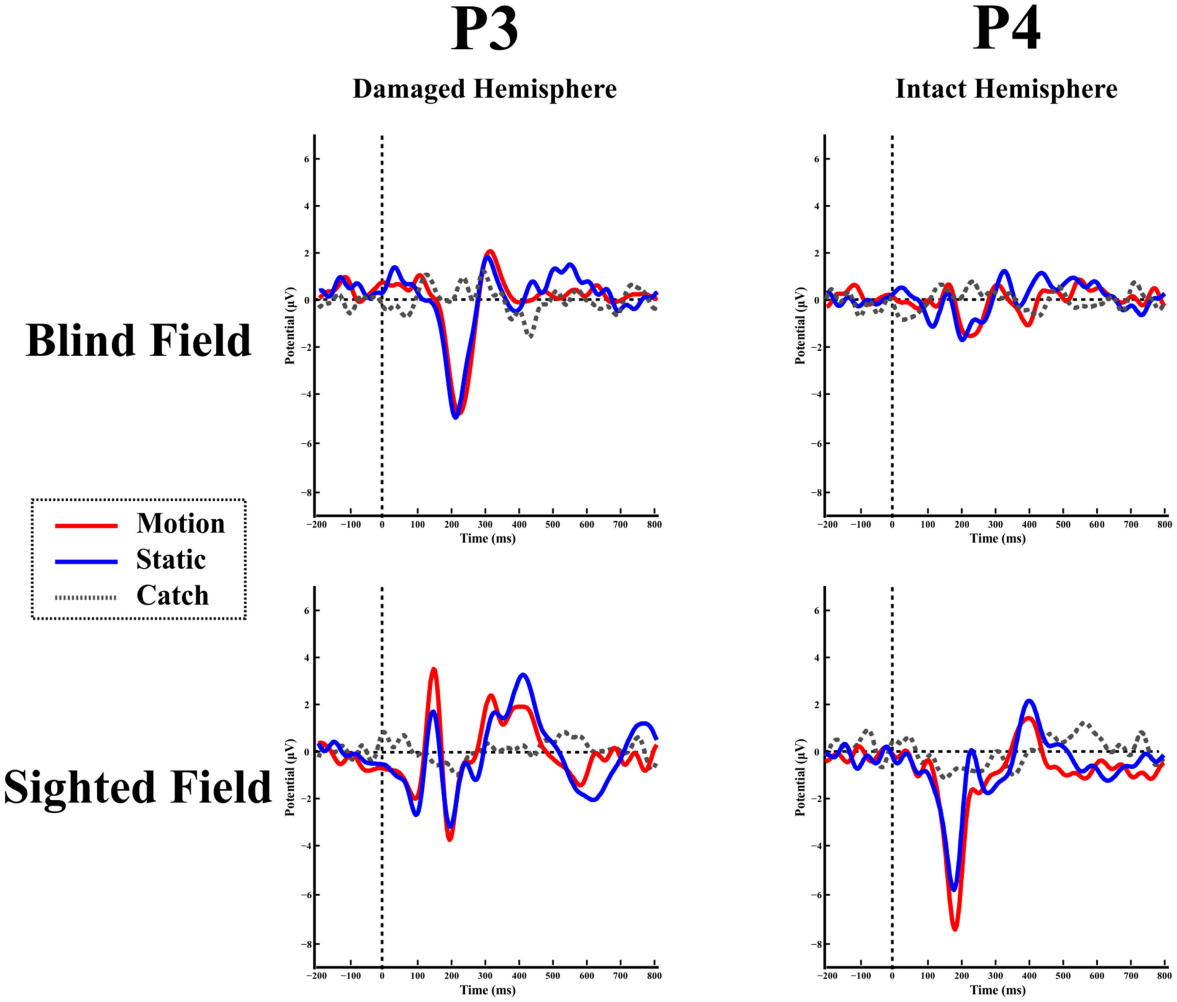
**Patient SL:** ERPs in the blind **(top)** and sighted field **(bottom)** as recorded at P3 (left-damaged hemisphere) and P4 (right-intact hemisphere) for the three stimulation condition.

**Blind hemifield**. Figure 5 shows the ERP responses as recorded at electrode P3 (damaged hemisphere) and P4 (intact hemisphere) for the two hemifields and the three stimulation conditions. In contrast to LF, visual inspection shows an early prominent negative component (N1) immediately followed by a positive (P2a) peak and later on by a long lasting negativity. The N1 is more pronounced in the damaged hemisphere.

**Figure 6:**
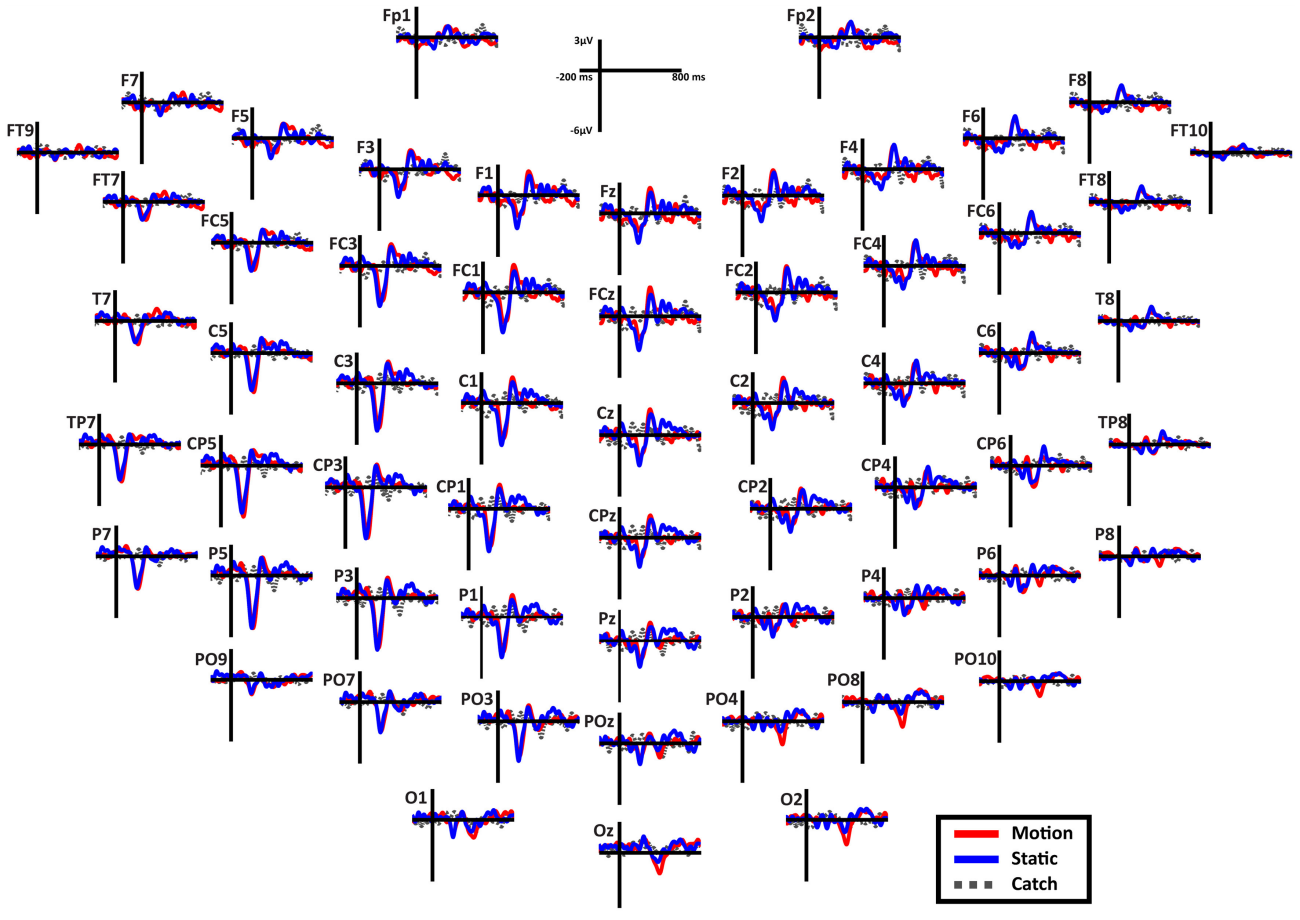
**Patient SL: Scalp distribution of the ERPs when the stimulus is presented in the blind field**. Note that the large negative peak (N1) is present in most electrodes in the left (damaged) hemisphere, while the frontal peak (P2a) is present in both hemispheres in the fronto-central electrodes. Finally, in the right posterior electrodes is present a late negativity selective for the motion stimuli.

**Overall scalp distribution**. Figure 6 shows the overall scalp distribution of responses for the two hemifields of SL. As mentioned above, a large negative peak (N1) is clearly visible between 180 and 260 ms after stimulus onset. It is present both for static and moving stimuli and is widespread across left hemisphere electrodes with a larger amplitude with respect to the right hemisphere. It is important to underline the absence of the early ERP components C1 and P1 and also of the later component P3. Immediately after the early negative frontal peak (N1) there is a positive component (P2a) visible in both hemispheres and a later component at posterior electrodes in the right hemisphere. Below we describe and analyze in detail these three components that index different aspects of the patient performance and subjective report.

**Figure 7:**
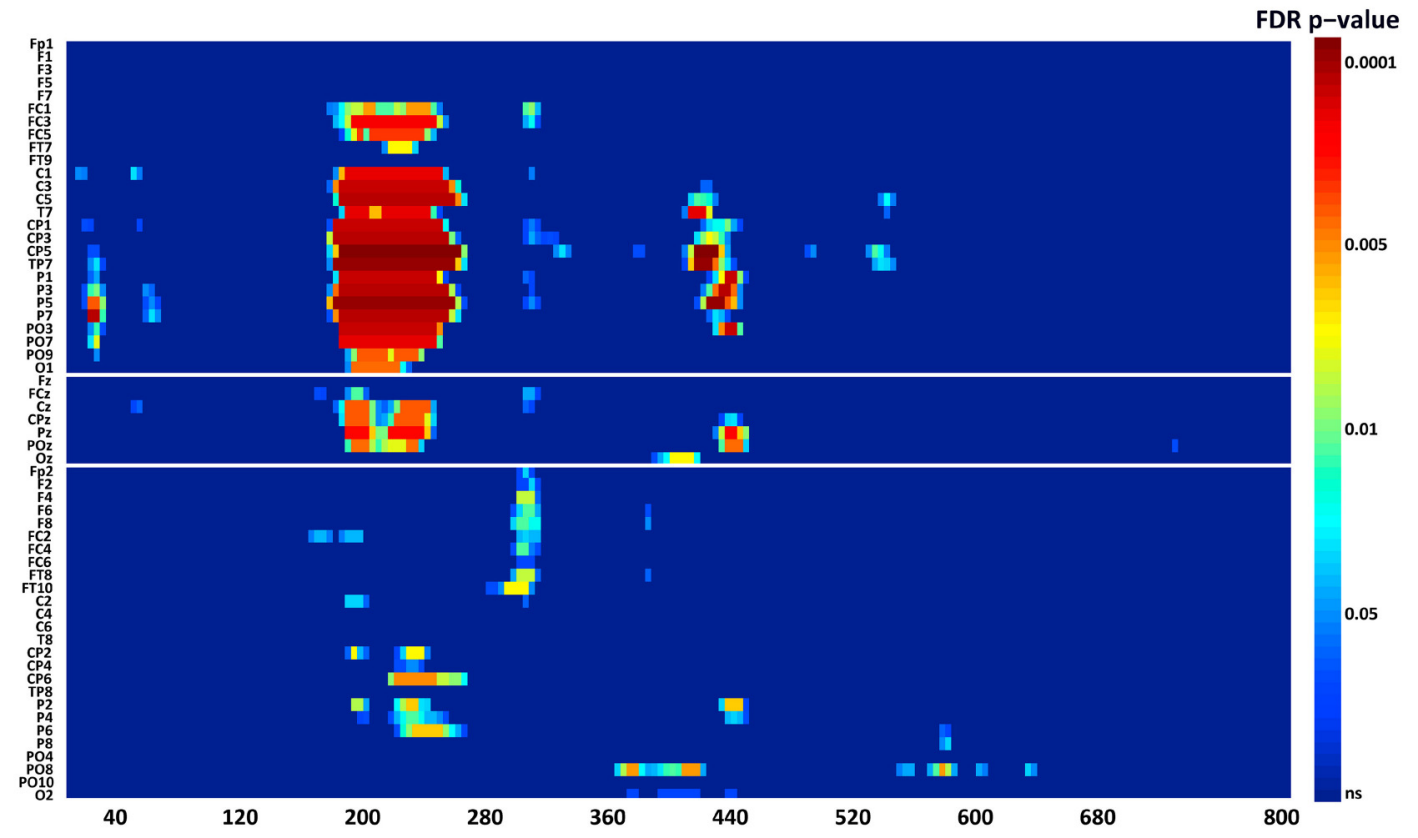
**Patient SL: Raster data resulting from the bootstrap ANOVA across all electrodes and the three conditions of blind field stimulation**. Color points represent the *p*-values after the FDR correction for multiple comparisons. Ordinates: left, electrode sites; right, *p*-values. Abscissae: post-stimulus onset time (ms).

**Figure 8:**
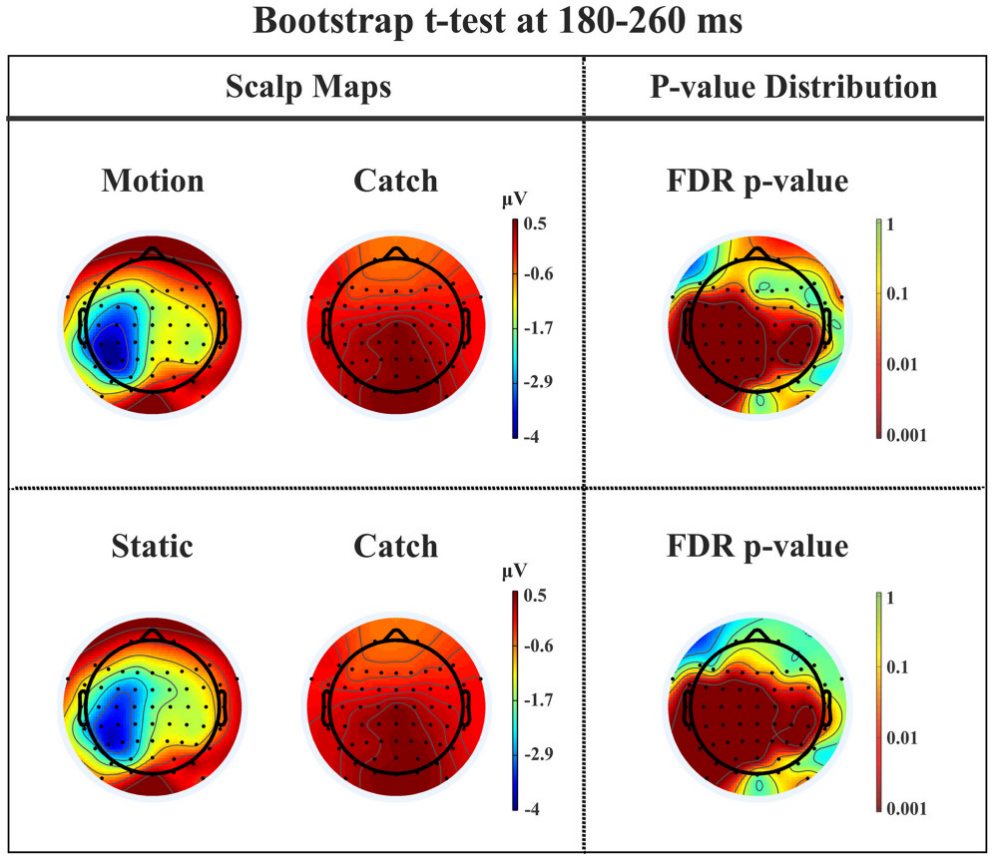
**Patient SL: Percentile Bootstrap re-sampling *t*-test between stimulus against no-stimulus conditions in the time window of the N1**. On the left side, Scalp maps representing motion against catch condition (upper panel) and static against catch condition (lower panel); Right side, distribution of the *p*-values after correction for multiple comparison.

**Figure 9:**
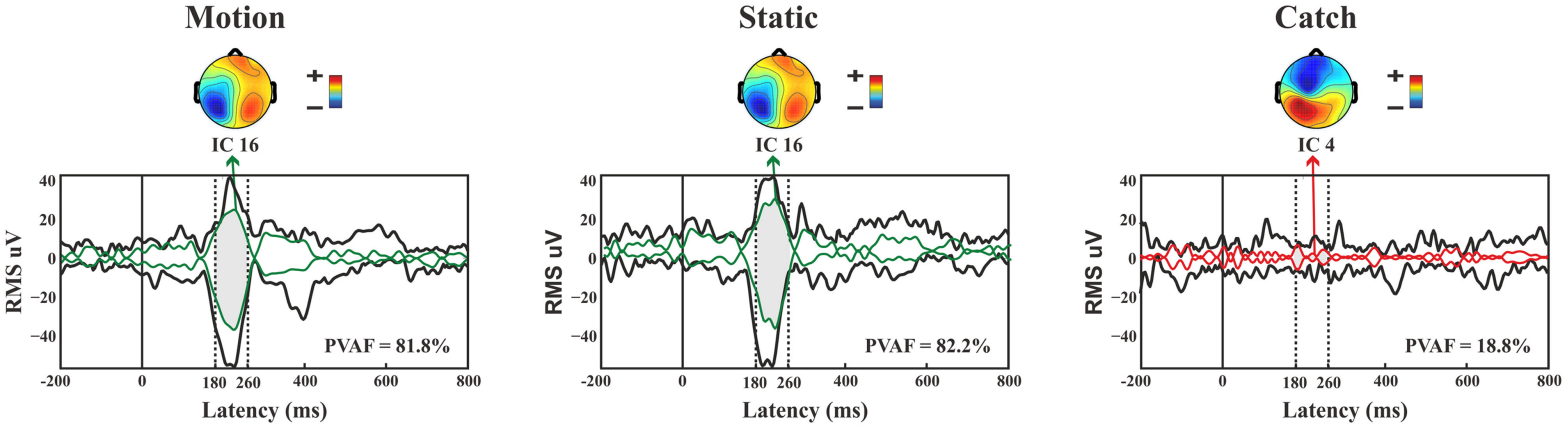
**Patient SL: Envelope of the ERP (black line) for the three conditions of stimulus presentation to the blind field**. The green line represents the contribution of the most prominent IC when the stimulus, either static or moving is presented. The red line represents the contribution of the most prominent IC during catch trials. The dotted lines represent the time window considered for the PVAF.

*N1*. A bootstrap ANOVA was conducted for the three conditions of stimulus presentation (static, motion and catch). The main results are shown in Figure 7: Significant FDR corrected *p*-values ranged between 0.00084 and 0.04977. As shown by the raster plot, the main difference between stimulus present and catch was in the N1 domain, in particular in the left posterior channels. In order to assess the reliability of the N1 peak two bootstrap *t*-tests were conducted: Moving vs. catch condition and static vs. catch. As can be observed in Figure 8 the results of the two tests were very similar (all significant FDR *p*-values ranged between 0.00042 and 0.04430). An important point is that the N1 component was mainly present in the ipsilesional electrodes, i.e., contralateral to the blind hemifield in the damaged hemisphere, see Figure 6. In order to examine its origin the PVAF (i.e., what percent of the scalp signal is reduced when a specific independent component IC is removed) was calculated from the ERP envelope for each condition of stimulus presentation (Figure 9). The result of this analysis showed that IC16 accounted for more than 80% of the ERP variance in that window; for the moving condition the PVAF was 81.8% with the maximum of variance at 224 ms; for the static condition was 82.2% with the maximum at 220 ms.

**Figure 10:**
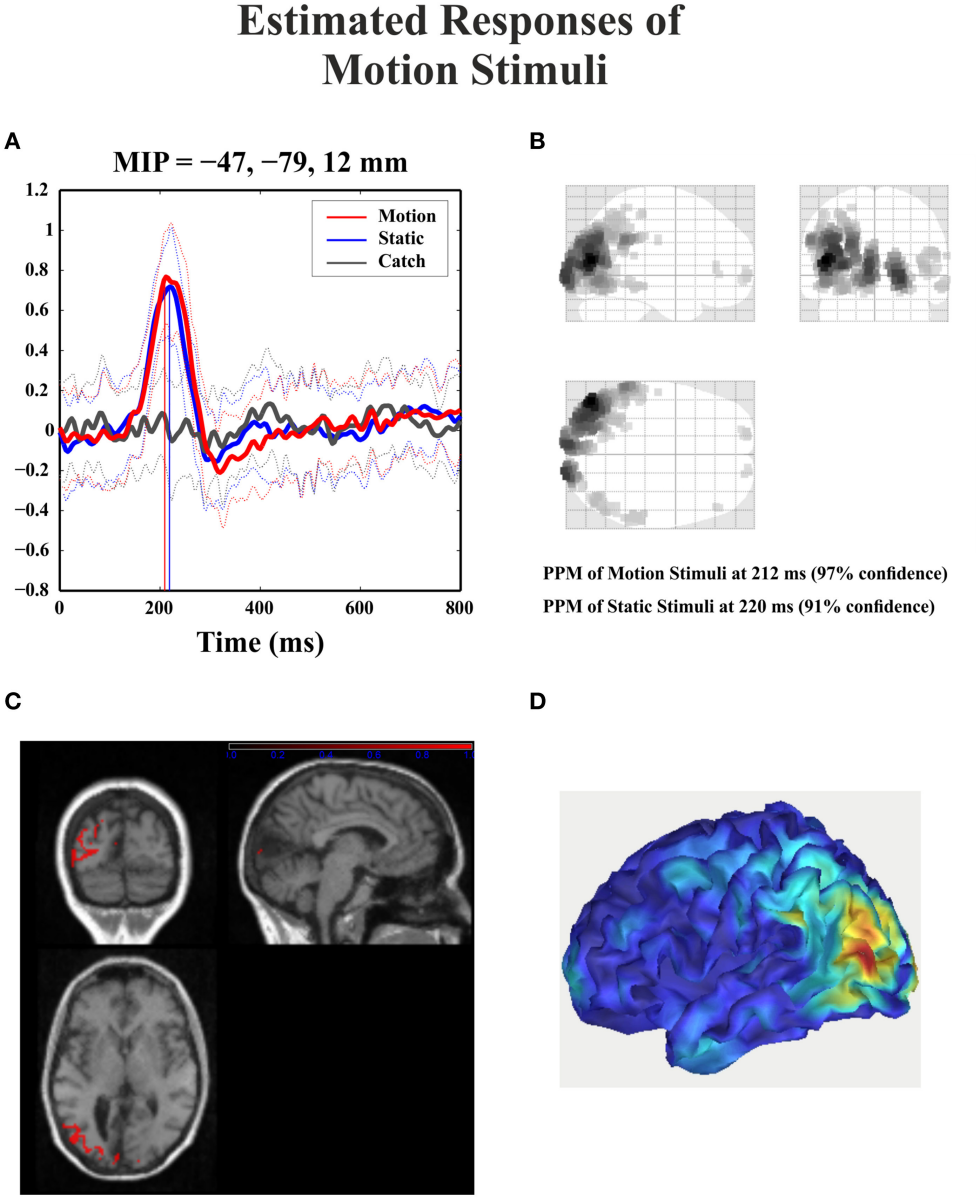
**Patient SL: 3D source reconstruction of the ERPs when the stimulus was presented in the blind hemifield. (A)** Time course of the region with maximal activity for the three conditions. For both motion and static stimuli the MIP is at the same time (corresponding to N1 latency) in the extra-striate cortex (BA19). **(B)** MIP of the 512 greatest source strengths within MNI space projected onto a glass brain for the motion condition. The area at the highest density correspond to left BA 19. **(C)** MIP of the statistical map for the motion condition projected on the T1-weighted images of patient SL showing both the lesion and in red the source reconstruction. **(D)** Summary power image from source reconstruction of motion stimuli presentation to the blind hemifield on a 3D rendered image.

For the catch condition the IC16 accounted only for the 4.4% of the variance while the IC with the highest PVAF accounted for the 18.8% (IC4). Thus, IC16 was specifically involved in the generation of the N1 peak following stimulus presentation. Furthermore, we conducted a 3D source reconstruction in the time window of the peak. In this window the MIP was at MNI coordinates (−47, −79, 12), i.e., in the left extra-striate area, BA19, see Figure 10.

**Figure 11:**
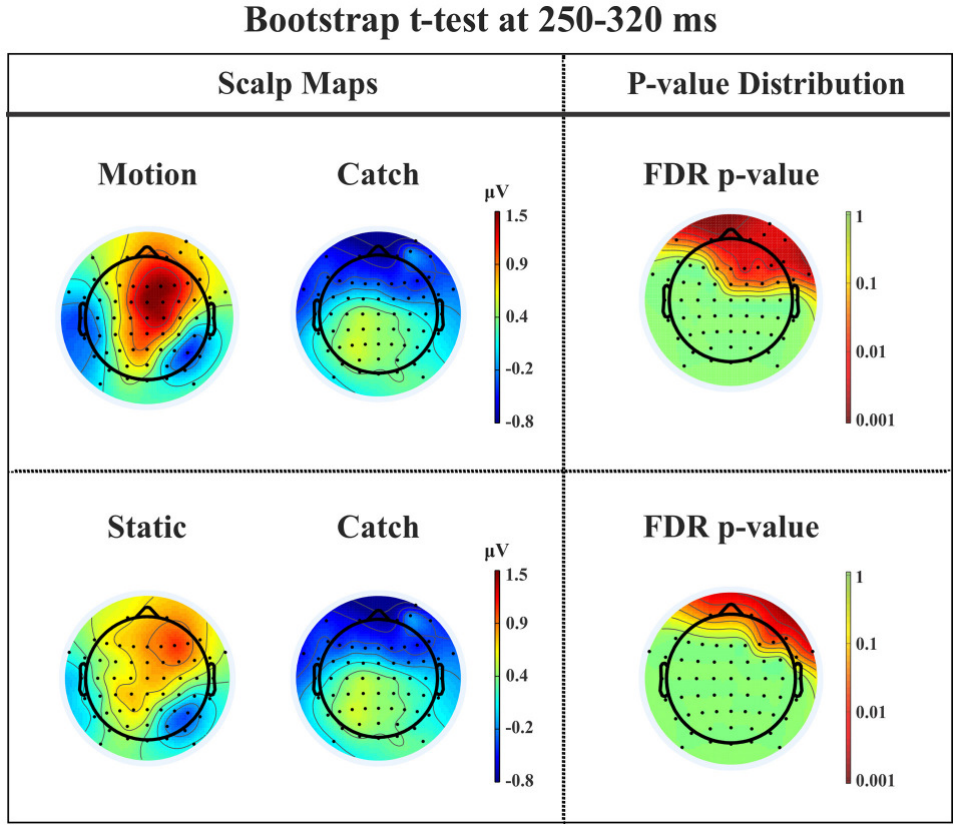
**Patient SL: Percentile Bootstrap re-sampling *t*-test between stimulus against no-stimulus conditions in the time window of the P2a**. On the left side, Scalp maps representing the motion against catch conditions (upper panel) and static against catch conditions (lower panel); Right side, distribution of the *p*-values after correction for multiple comparison.

*P2a*. At left fronto-central electrodes a significant positive peak was found immediately after N1, with a small amplitude and a latency between 250 and 320 ms after stimulus onset. It was present for both moving and static stimuli (the significant FDR corrected *p*-values for the motion condition against the catch ranged between 0.0031 and 0.0443 and those for the static condition ranged between 0.0061 and 0.0351), see Figure 11.

This component has a spatio-temporal distribution similar to a positive component referred to in the literature as anterior P2 (P2a: Potts et al., 1996; Potts and Tucker, 2001; Brignani et al., 2009) or frontal P3 (P3f: Makeig et al., 1999) and also as frontal selection positivity (FSP: Kenemans et al., 1993; Martens et al., 2006). Indeed, its latency between 200 and 300 ms after stimulus onset, a positivity distribution in the frontal electrodes, and the fact that its peak emerges immediately after the N1 are similar to the typical characteristics of the P2a. Moreover, we found that this component was significantly different from catch trials for both motion and static stimuli.

**Figure 12:**
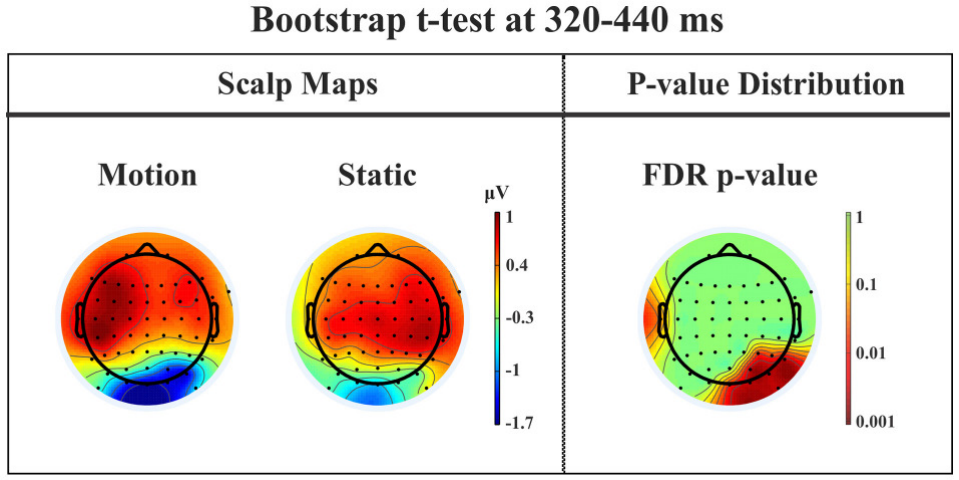
**Patient SL: Percentile Bootstrap re-sampling *t*-test between Blind Motion and Blind Static conditions in the time window from 320 to 440 ms**. On the left side, Scalp maps representing the Bootstrap *t*-test to compare the motion against static; on the right side the distribution of the *p*-values after the correction for multiple comparison.

*Late posterior negativity*. Finally, an additional bootstrap *t*-test was conducted between static and moving trials in the time range of 320–440 ms (Figure 12). This time window was chosen because of the presence of a negative peak in right posterior electrodes that was present only for the moving condition. The results showed a significant difference between static and motion conditions for posterior right channels (P4, P6, P8, PO4, PO10, and Oz) as well as T7 in left hemisphere (significant FDR corrected *p*-values ranged between 0.0024 and 0.0175). The envelope in this time window, see Figure 9 showed that the IC accounting for most of the variance was IC16 with PVAF of 49.4% in the moving condition, while in the static condition its contribution was negligible (PVAF −3%).

**Intact hemifield**. The patient's ERPs when the stimulus was presented in the intact hemifield were similar to healthy participants in latency as well as in amplitude (Figure 5 bottom panel). Notice the presence of a large P3 component in the contralateral hemisphere. The difference between the responses from the impaired and unimpaired hemisphere are similar to responses from the two hemispheres in the control group as demonstrated by the RSDT test, where no pairs of electrodes of SL resulted different from controls (all *p*-value > 0.05).

## Discussion

### N1-P2a components

As described in the Results, patient SL upon stimulus presentation in the blind hemifield reported that she had no clue as to the features of the stimuli, i.e., about their orientation or whether they were static or moving. However, she was consistent in discriminating catch from stimulus trials, as reported in preliminary testing and in another study (Mazzi et al., 2016), as well as after each trial block of the present study, in reporting the occurrence of the stimuli as “something appearing in the visual field.” In contrast, the other hemianopic patient LF never experienced the presence of the stimuli or a difference between catch and stimulus trials. An important finding is a clear correspondence between the subjective reports of the two patients and their electrophysiological responses to stimuli presented to the blind field. Patient LF did not provide any perceptual report while in SL two ERP components could be considered as likely correlates of her report, namely N1 and P2a. Both components were present irrespective of whether the stimuli were static or moving and therefore cannot be considered as related to the behavioral evidence of a static-motion discrimination but rather to the “feeling that something appeared in the blind field”. The time window of the N1-P2a components is roughly compatible with that of the Visual Awareness Negativity (VAN) that is, a ERP component resulting from the difference between conscious and unconscious stimulus processing (Koivisto and Grassini, 2016; Koivisto et al., 2016). Notably, the electrode location where the N1 was clearly detectable was widespread in the ipsilesional hemisphere, see Figure 6. Its source could be located mainly in the extrastriate cortex (BA 19) of the left and, to a lesser extent, of the right hemisphere. It is important to underline that, in contrast to N1, the early components, C1 and P1, and the later component, P300, were absent following blind field stimulation. The absence of C1 and P1 might explain the incapacity of SL to discriminate the orientation of static stimuli and is a likely consequence of the striate cortex lesion impairing initial basic sensory processing with static stimuli. In healthy subjects, the N1 was followed by a small amplitude complex P2-N2 and by a very large P300 while this was not the case in SL. This is in keeping with her lack of full stimulus awareness. Thus, we believe that the presence of N1 and P2a might be considered as an electrophysiological correlate of degraded conscious vision. This possibility is reinforced by the source of N1 in BA 19 which is broadly in agreement with Bagattini et al., (2015), Koivisto and Grassini (2016) and Tagliabue et al. (2016), and with the hypothesis of an early site of emergence of perceptual awareness. In the present case, however, it is awareness of degraded rather than full vision. The relationship between N1 and perceptual awareness is controversial. On one hand N1 has been repeatedly associated with selective attention, see Mangun (1995), rather than with consciousness. In keeping with that, Sergent et al. (2005) in an attentional blink paradigm found that the presentation of unseen words yielded a P1 and N1 component prior to emergence of consciousness that occurred at later processing stages. On the other hand, there is evidence for a link of N1 with awareness. For example, in a face inattentional blindness paradigm Shafto and Pitts (2015) found that the N170 was present only in the aware condition. Studies of binocular rivalry have also provided important information on the physiological correlates of consciousness. For example, Kaernbach et al. (1999) and Roeber and Schröger (2004) have shown that changes of perceptual awareness are witnessed by changes of the N1 component and this is in accord with an early emergence of consciousness probably made possible by feedback processes involving V1, see Di Lollo et al. (2000), Lamme and Roelfsema (2000), and Tong (2003). Thus, although debated, the involvement of N1 in an early onset of perceptual awareness seems to have solid grounds.

### Late posterior negativity

**Figure 13:**
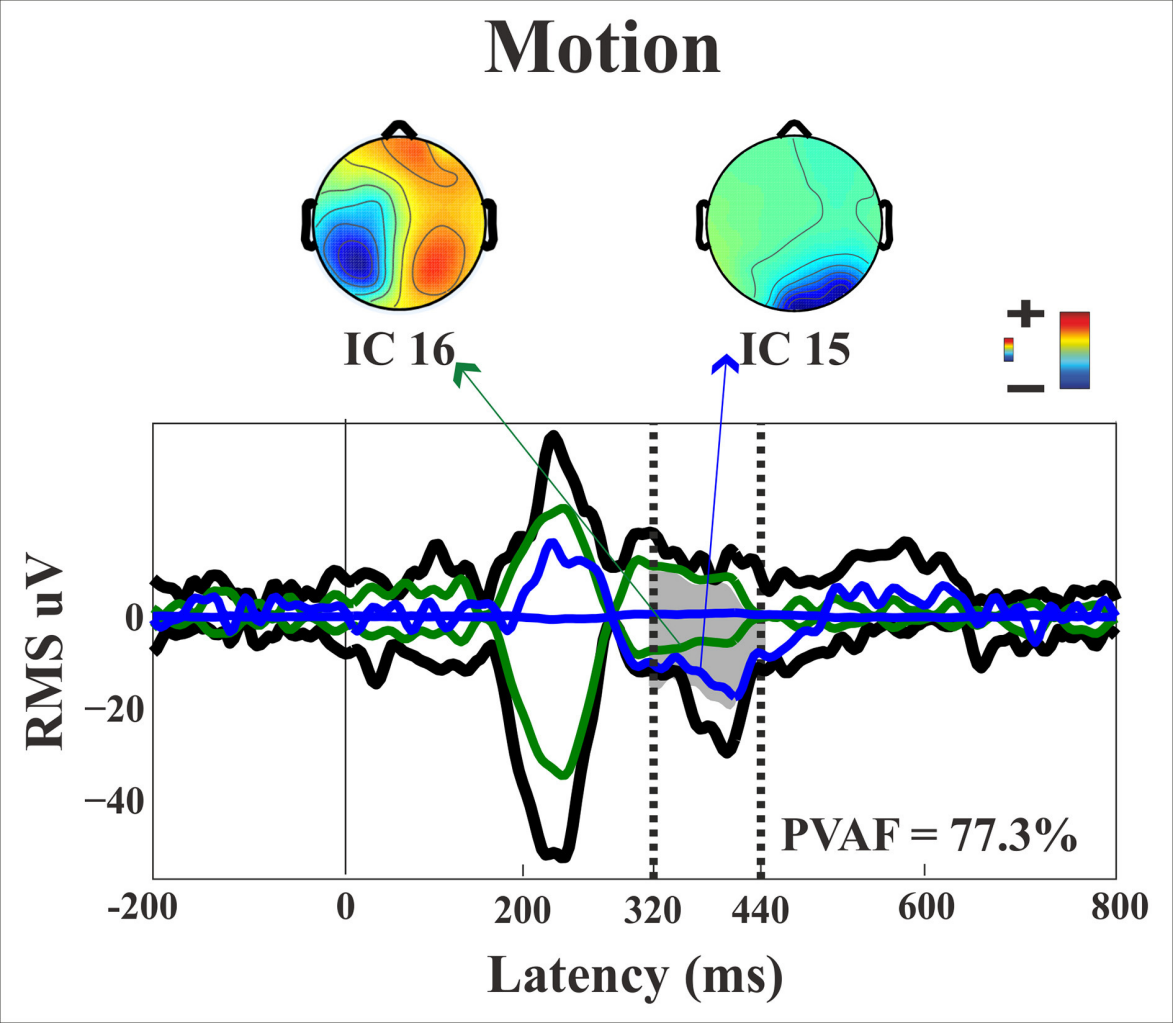
**Patient SL: Envelope of the ERP (black line) for the motion condition of stimulus presentation to the blind field**. The green and blue lines represent the contribution of the two most prominent ICs. The dotted lines represent the time window considered for the PVAF.

Starting from the historical finding by Riddoch (1917), who provided evidence of residual degraded vision for moving stimuli in cortically blind patients (Zeki and Ffytche, 1998), that motion stimuli are the most frequent protagonists of above chance unconscious discrimination (blindsight type 1) is a well-established notion, see Ajina and Bridge (2016) for a recent review and Azzopardi and Cowey (2001) for controversial evidence. In the present study, the novel finding is that the presence of motion made possible the above-chance discrimination of another visual feature, namely pattern orientation. Patient SL was able to discriminate orientation only when the gratings drifted either horizontally or vertically. This effect might be attributed to activity of cortical motion area V5/MT receiving input from subcortical centers bypassing V1 (Ajina et al., 2015a; Kavcic et al., 2015) and retaining the capacity of discriminating apparent motion in the absence of V1. Importantly, the ERP results in patient SL showed a difference between the static and the motion condition in the posterior electrodes of the intact right hemisphere, see Figure 13, as a negative peak around 390 ms post stimulus onset. The PVAF analysis showed that this peak could be accounted for by the same ICs as for the N1 but bilaterally distributed and is in agreement with V5/MT activity. Thus, moving stimuli engage large neuronal pools that enable an effective interhemispheric transfer of directional movement information presumably at parietal level. In keeping with this possibility is the finding in the present study of ERP differences between moving and static stimuli in the right hemisphere of healthy controls. In addition, these results are in accord with Kavcic et al. (2015) who found an interhemispheric transfer of motion information in hemianopic patients with left but not right hemisphere damage. Indeed, one likely possibility is that the blindsight exhibited by patient SL might be subserved by the intact (right) hemisphere as a result of interhemispheric integration and this is in broad agreement with the results of Celeghin et al. (2015a) who found that in hemianopic patients the above-chance visuo-motor responses in a simple RT paradigm depended on the intact hemisphere as a result of interhemispheric transfer. This is in broad keeping with Silvanto et al.'s (2007) results on hemianopic patient GY. They found that he experienced visual phosphenes in the blind hemifield following bilateral transcranial magnetic stimulation (TMS) of area V5/MT, while this was not the case with unilateral stimulation on the damaged hemisphere. This is clearly in support of a crucial contribution to the emergence of a form of visual awareness in an otherwise blind visual field.

## Conclusion

In conclusion, we found that a hemianopic patient with a selective lesion of left V1 showed an above-chance discrimination of the orientation of moving visual gratings presented to the blind hemifield. Importantly, the patient reported no visual experience of the different features of the stimuli but a visual sensation that something appeared in the blind hemifield. This subjective observation found an electrophysiological correlate in the presence of a N1 and of a frontal P2a component. In contrast, the earliest visual components such as C1 and P1 and the later P300 could not be identified.

Thus, as to the earliest physiological correlate of perceptual awareness our results support an early stage occurrence. However, this conclusion applies to a form of degraded visual experience and might not necessarily be generalized to onset of full perceptual awareness.

As far as the blindsight effect found for the discrimination of the orientation of moving stimuli is concerned, a very likely physiological correlate is represented by the posterior late negative component in the intact right hemisphere. It is important to reiterate that the behavioral performance of patient SL can be classified as a form of blindsight made possible by stimulus motion. This behavior is independent from the perceptual awareness experienced by the patient in so far as she reported the same degraded visual sensation for both static and motion stimuli, while the unconscious above-chance performance emerged only in the motion condition.

Finally, one should note that patient SL underwent a stroke about 6 years before the present testing and that this suggests the possibility of plastic neuronal reorganization of her cortical and subcortical areas. It would be interesting to gather further information in future testing sessions to find out whether this reorganization is still in progress and might further enable a shift from totally or partially unconscious behavior to full perceptual awareness.

## Author contributions

AB conducted the research, analyzed the data, and drafted the manuscript. JS conducted the research and collaborated to data analysis. SS discussed the data and revised the manuscript. CM discussed the data and revised the manuscript.

## Funding

This research was supported by ERC Grant 339939 Perceptual Awareness (PI: CM).

### Conflict of interest statement

The authors declare that the research was conducted in the absence of any commercial or financial relationships that could be construed as a potential conflict of interest.

## Footnotes

^1^MATLAB version 8.2.0 (R2013b) Natick, Massachusetts: The MathWorks, Inc., 2010.
